# A single nucleotide mutation in *Nppc *is associated with a long bone abnormality in *lbab *mice

**DOI:** 10.1186/1471-2156-8-16

**Published:** 2007-04-17

**Authors:** Yan Jiao, Jian Yan, Feng Jiao, HongBin Yang, Leah Rae Donahue, Xinmin Li, Bruce A Roe, John Stuart, Weikuan Gu

**Affiliations:** 1 Departments of Orthopaedic Surgery – Campbell Clinic and Pathology, University of Tennessee Health Science Center (UTHSC), Memphis, Tennessee, USA; 2 Department of Medicine, UTHSC, Memphis, Tennessee, USA; 3 The Jackson Laboratory, Bar Harbor, Maine, USA; 4 Functional Genomics Facility, University of Chicago, Chicago, Illinois, USA; 5 Department of Chemistry and Biochemistry, University of Oklahoma, Norman, Oklahoma, USA

## Abstract

**Background:**

The long bone abnormality (*lbab*) mouse is a new autosomal recessive mutant characterized by overall smaller body size with proportionate dwarfing of all organs and shorter long bones. Previous linkage analysis has located the *lbab *mutation on chromosome 1 between the markers *D1Mit9 *and *D1Mit488*.

**Results:**

A genome-based positional approach was used to identify a mutation associated with *lbab *disease. A total of 122 genes and expressed sequence tags at the *lbab *region were screened for possible mutation by using genomic DNA from *lbabl/lbab, lbab*/+, and +/+ B6 mice and high throughput temperature gradient capillary electrophoresis. A sequence difference was identified in one of the amplicons of gene *Nppc *between *lbab/lbab *and +/+ mice. One-step reverse transcriptase polymerase chain reaction was performed to validate the difference of *Nppc *in different types of mice at the mRNA level. The mutation of *Nppc *was unique in *lbab/lbab *mice among multiple mouse inbred strains. The mutation of *Nppc *is co-segregated with *lbab *disease in 200 progenies produced from heterozygous *lbab*/+ parents.

**Conclusion:**

A single nucleotide mutation of *Nppc *is associated with dwarfism in *lbab/lbab *mice. Current genome information and technology allow us to efficiently identify single nucleotide mutations from roughly mapped disease loci. The *lbab *mouse is a useful model for hereditary human achondroplasia.

## Background

Annually, approximately 2.3% of infants in the United States are born short (small) for gestational age (SGA) [1]. Achondroplasia (short-limbed dwarfism) is the most common cause of human dwarfism [2], and it accounts for 70% of dwarfism cases [3] To explore causative genetic factor(s) for the development of achondroplasia in animal models, we focused on a novel spontaneous model of long bone abnormality called *lbab *mouse, which was originally found in the PL/J strain at The Jackson Laboratory (TJL)[4]. Homozygous mutants exhibit proportionate dwarfing of all organs and shorter long bones. The mutation has been transferred to the C57BL/6J strain to improve reproduction. The genetic locus responsible for the phenotype has previously been mapped to chromosome 1 (Chr 1) between markers *D1Mit9 *and *D1Mit488 *(53.5 cM) at TJL [4], but the responsible gene and the nature of mutation remained unclear.

Disease gene hunting has always been time-consuming and labor-intensive. For successful map-based cloning, a complicated fine mapping of a major locus is generally essential [5]. Recently, we developed an alternative, sequence-based, positional candidate cloning approach to bypass this bottleneck of cloning, and we have successfully identified several mutated genes in different mouse spontaneous mutants by applying this strategy [6,7]. Our strategy takes advantage of the availability of comprehensive murine sequence databases, polymerase chain reaction (PCR), high throughout PCR product analysis, and sequencing technologies to speed up the process of disease-related gene hunting. Interestingly, we identified a nucleotide mutation (C→G transversion) in gene *Nppc *in the *lbab *mice. Herein, we describe the detailed process of our cloning and validation.

## Results

### Body growth of *lbab/lbab *mice

**Figure 1:**
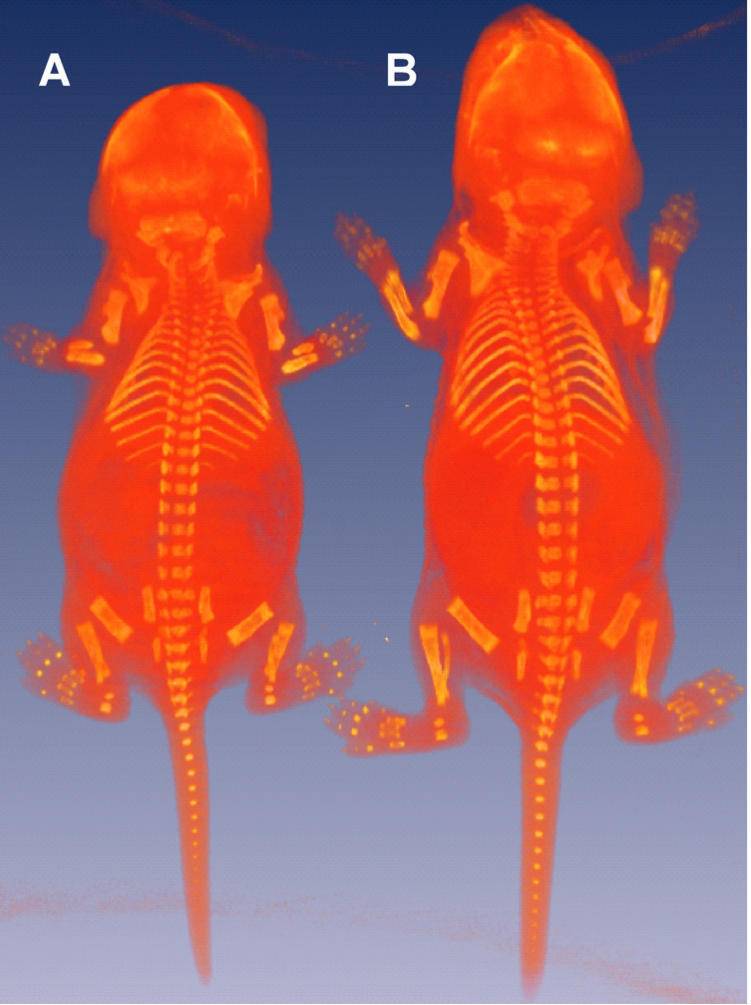
**Phenotypic assay of normal and *lbab *mice**. A whole-body microCT scanned image of a pair of *lbab/lbab *and +/+ B6 mice in the same litter at the age of 6 days. **A: ***lbab/lbab *mouse, **B: **+/+ mouse.

All *lbab/lbab *mice housed at the University of Tennessee Health Science Center died before 7 days of age with remarkable changes in organ weight and body size (Fig. 1, Table 1). No detectable differences in survival rate or body size were noted between the *lbab/+ *and +/+ mice.

**Table 1:** Comparison of phenotypic parameters between mice with different genotypes at the *lbab *locus

Parameter	+/+ (n = 10)	*lbab*/+ (n = 10)	*lbab/lbab *(n = 12)	*lbab/lbab *as % of +/+
Body weight (g)	3.45 ± 0.422^1^	3.61 ± 0.351	1.54 ± 0.255*	44.1%
Body length (mm)	55.9 ± 0.36	56.7 ± 0.34	34.0 ± 0.35*	61.6%

^1 ^Values were mean ± SD. Mice of different gender were pooled as they were too young to show detectable differences; +/+ mice included 5 females and 5 males, 10 *lbab*/+ mice included 5 females and 5 males; 12 *lbab/lbab *mice included 6 females and 6 males. As shown, the body weight and body length from the *lbab/lbab *group were significantly smaller than in the +/+ and *lbab*/+ groups (* p < 0.05). There were no significant differences between +/+ and *lbab*/+ mice.

### Target region of the mutation in *lbab *locus

**Figure 2:**
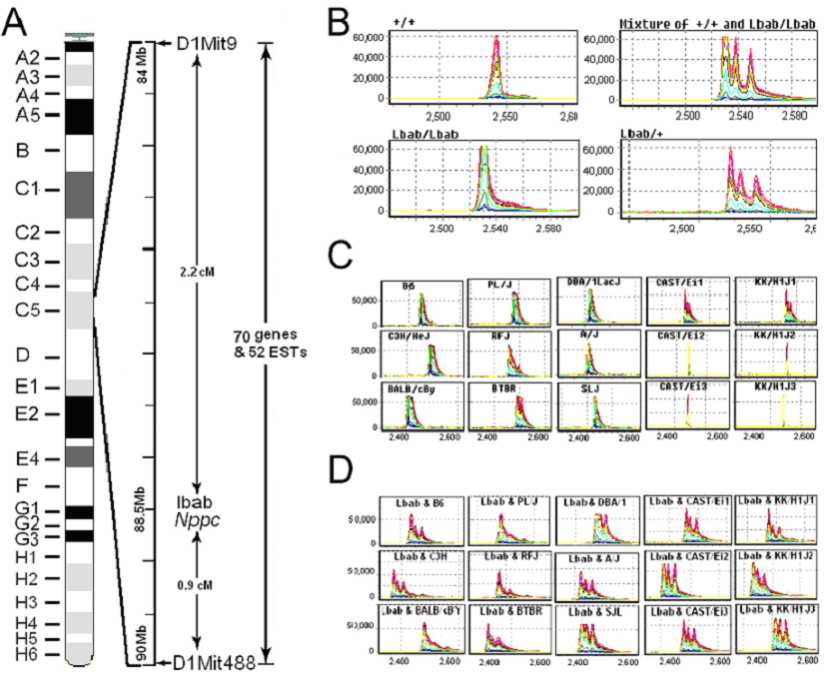
**Schematic of the mutation identification in *lbab *mice**. **A: **A genetic map of the *lbab *locus showing the relative locations of microsatellite markers and the total number of candidate transcripts within the *lbab *locus. **B: **PCR product analyses using the SpectruMedix system. The PCR products from different species of mice were pair mixed to check the possible sequence difference between normal B6 and PL/J mice (+/+), normal and homozygous *lbab *mice (mixture of +/+ and *lbab*/*lbab*), intrahomozygous *lbab *mice (*lbab*/*lbab*), and intraheterozygous mice (*+/lbab*). **C**: The PCR amplification products of exon 2 of the *Nppc *gene from 11 strains of mice (C57BL/6J, SJL/J, CAST/Ei/J, C3H/HeJ, PL/J, BALB/cJ, RF/J, KK/H1J, A/J, DBA/1J, and BTBR/J). **D**: PCR products from each of those strains were mixed separately with that of *lbab/lbab *mice. Each mixture showed multiple bands of signal, indicating the difference in their DNA sequences. The X-axis represents relative size of the PCR products. The Y-axis represents relative strength of signal or the amount of the PCR products.

Previous genetic analysis showed that the *lbab *mutation is located on mouse Chr 1 and is flanked by molecular markers *D1Mit9 *and *D1Mit488 *[4]. According to the Ensembl database, *D1Mit488 *is located between 91902920–91903043 bps. However, there is no physical position for *D1Mit9 *in the database, although we know from TJL database that it is positioned at 53.5 cM. From TJL's mouse genome informatics database, we found 13 molecular markers at the 53.5 cM position. The positions of most of these [8] markers are near 84 Mb [9]. Accordingly, we decided to start our investigation in the region between 83 and 90 Mb (Fig. 2A). Genomic sequences within this region are complete in the Ensembl database. There are a total of 122 transcripts, 70 of which represent genes and 52 represent expressed sequence tags (ESTs) (Table 2).

**Table 2:** Distribution of candidate transcripts around the *lbab *locus

Region	Gene	EST
83–84	14	4
84–85	3	5
85–86	1	6
86–87	22	13
87–88	20	11
88–89	10	6
89–90	0	7
Total	70	52

### Initial screening of the targeted region

Because *lbab *mice are bred on a C57BL/6J (B6) background, we assumed that the majority of the background genome carrying the *lbab *mutation was from the B6 strain. Therefore, we isolated genomic DNA from both B6 and *lbab/lbab *mice. We designed 528 pairs of primers flanking first and last exons of 122 candidate transcripts by using Primer3 software[10]. We obtained the primers commercially (Illumina, San Diego, CA) and conducted PCR amplification with genomic DNAs from *lbab/lbab *and B6 mice, which were regarded as normal controls. The PCR products were then analyzed for the presence of sequence differences between *lbab/lbab *and normal mice by using the RevealTM system (SpectruMedix LLC, PA). We found variations in 25 PCR products between normal B6 and *lbab/lbab *mice (Table 3).

**Table 3:** Primers flanking polymorphism sites between C57BL/6J and *lbab/lbab *mice

Gene ID (Ensembl)	Genomec Position	Forward Primer's Sequence	Reverse Primer's Sequence
ENSMUSG00000036707	86006371–86006887	GATGGGATGCAGTGTGTCAG	AAACTGCAGGCGCACTAAAC
ENSMUSESTG00000001751	83966699–83966785	CAGCAGAAGTGAAACCAGGA	TGACTCTGAGCTCACCATGC
ENSMUSG00000026163	84087634–84088853	GGGATGAACAGCATTTGAGA	TGCTCCCTGGGAAGTAACAG
ENSMUSESTG00000001946	85602128–85646987	TGATTCCCAACTCCTTTTCAG	GGCAATACACCTTGCTACTCC
ENSMUSG00000026220	85713718–85742232	ACATGCCTGGCTTGAAAGTT	GGATGGATCTTTGACATCACC
ENSMUSG00000026200	85713718–85742232	GAAGTGGGTGAGTTGCAGTG	GCAAGAGGGTCAGAGGTTCA
ENSMUSESTG00000001946	85877547–85896383	ACTGGAGCTCTGGTGTCCAT	GGAAGGGACAAACTCTTCCA
ENSMUSESTG00000001946	85877547–85896383	TCCCTCACTTCATCCCAAAC	CCAACAACTTGCTCCATTGTAA
ENSMUSESTG00000001946	85877547–85896383	GCATGGAGTCAGCTGTGCTA	GCTGTGCAGTGATCAGCAGT
ENSMUSG00000026228	86273443–86274025	TGTTCAGTGGCAGGAACAAG	GAGTCACCAAAAGGCGAATG
ENSMUSESTG00000004625	86885787–86885885	GCTTCGCTCTTGGCTCAAC	TCCTCTCGCACTGAACTGAA
ENSMUSESTG00000004625	87005019–87005248	GGCAGTTAAACCAGAGAGCA	GCCCAAGACTCACCTCTGTC
ENSMUSG00000036511	87288388–87288749	AGTTGCAAGGACAGCAGGAC	GCCTAGCTTGAGGTGCTGAC
ENSMUSG00000026253	87397283–87403022	AGGCTGACACATGACACTCC	CCCCAGGTCTCAAATCCCTA
ENSMUSG00000026254	87405306–87405408	ATCCACGGGGAGGTTAAAGT	ACGCGTCGAACTTGTTGTTC
ENSMUSG00000026254	87411304–87411418	CTTGGGCTATGACCTTGTGG	CCTGTATCATCACCCCAAAC
ENSMUSG00000026254	87417820–87417956	TCACTCTGTTCCTCCCTTAGGA	GGCCCAACTATGGAGCTACT
ENSMUSG00000026255	87484369–87484561	TCCTTGGACCATGCAGGTAT	TATCCAACCAGGGAGCATTC
ENSMUSG00000026255	87504768–87505947	CGACCCCATTCAAATCAGAG	TCAGAAGCCCAATCCTGAAC
ENSMUSG00000044104	87581463–87582114	GCTGCATAGCTGGCTGCATA	AGAAGGCACTGGGGAAATCT
ENSMUSG00000048000	87641410–87641794	ACAAGGCTGGAGTTGCTCTC	AGCCTCAAGAGAGGCGAGTA
ENSMUSG00000026259	87686025–87686185	GTGCTTACCGCTCACTGACA	GCTTCAGGACATCTGCCTCT
ENSMUSG00000026288	87914088–87914491	CTGAGGGCAGAATGACCAGT	CCCAGGTTTAGCCCTTCTTC
ENSMUSG00000026289	87985895–87986828	GGGAAAGCATTCTCTGTCCT	GACAATCAAGACGCAAGGAG
ENSMUSG00000026289	87985895–87987101	AGGCTTTGTACGCAGACTCC	GCACAAGTTGGTTTGGGACT

We speculated that the identified sequence variations were likely to arise from the close linkage between the mutated gene and nearby sequences in the parental PL/J strain, even though the background of *lbab *mice was mainly on the B6 strain. To determine if this were the case, we isolated DNA from PL/J mice obtained from TJL and amplified the 25 variable fragments by using the same panel of primers. By comparing those DNA fragments with those from *lbab/lbab *mice, we did not find sequence differences between DNA products from *lbab/lbab *and PL/J mice (data not shown), suggesting that those 25 variations represent polymorphisms between PL/J and B6.

### Detecting the mutation in *lbab *locus in a more focused region

Because of the recognition of sequence polymorphisms between PL/J and B6 mice in the targeted region, we made two changes in our follow-up screening. First, we switched our controls from B6 to PL/J mice. Using another panel of primers (n = 240 pairs) for PCR amplification of every exon of all candidate genes, we identified only one DNA fragment from *lbab/lbab *mice that was different from PL/J mice DNA. This fragment was from exon 2 of the gene ENSMUSG00000026241, representing the gene for natriuretic peptide precursor C (*Nppc*). The same pair of primers for this fragment was used for further genotyping as indicated in material and methods.

**Figure 3:**
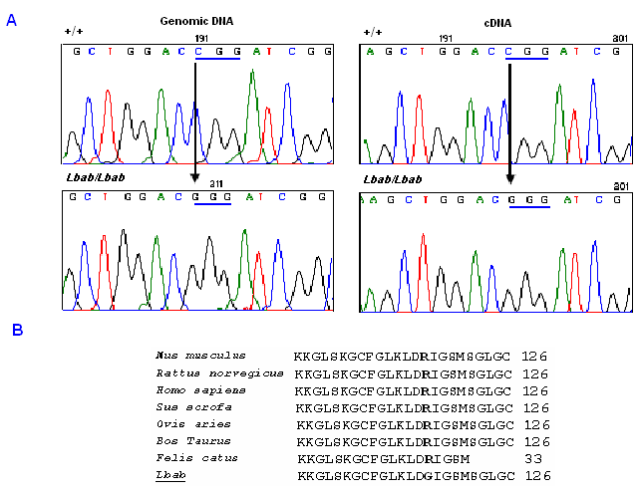
**Schematic of the sequence changes of *Nppc *gene in *lbab *mice**. **A**: A stretch of nucleotide sequence of *Nppc *in *lbab/lbab *and +/+ mice. A C→G transversion was found in both genomic DNA and cDNA sequences of *Nppc *in *lbab/lbab *mice compared with the sequence from PL/J mice. **B: **Alignment of the predicted amino acid sequence in the conserved domain of *Nppc *in different species. The C→G substitution leads Arg (R) to Gly (G) substitution at codon 117 on the conserved domain of *Nppc*.

Because we used PL/J mice as controls for this cycle of screening, there was another concern that the sequence variation might be a polymorphism derived from the B6 strain. To address this issue, we used PCR amplification of genomic DNA from B6 mice and compared results to the PCR products from PL/J and *lbab/lbab *mice. The data showed that the amplified DNA fragment from *lbab/lbab *mice was different from both B6 and PL/J mice, while the fragments from PL/J and B6 were the same (Fig. 2B). To find out which nucleotide(s) was different between *lbab/lbab *and PL/J controls, we sequenced genomic DNA fragments from PL/J and *lbab/lbab *mice. The data revealed a C→G change from PL/J to *lbab/lbab *mice (Fig. 3A, left panel).

To confirm the same difference at the cDNA level, we performed reverse transcriptase PCR (RT-PCR) on total RNAs from *lbab*/*lbab*, *lbab*/+, and +/+ mice by using primers that covered the mRNA sequence from exon 2 to exon 3 of the *Nppc*. The resultant RT-PCR products were sequenced using the SpectruMedix system, and the same C→G transversion from PL/J to *lbab/lbab *mice was found (Fig. 3A, right panel)

To evaluate the potential consequence of this point mutation in *Nppc*, we examined the translated amino acid sequence of *Nppc*. We found that this transversion predicts the substitution of arginine (R) for glycine (G) in a conserved domain of Nppc protein (Fig. 3B).

### Confirmation of mutation

We conducted further experiments to confirm the single nucleotide change in the *Nppc *gene. Based on our initial screening, we think that it is very likely that the C→G change in exon 2 of the *Nppc *gene is causally related to the phenotypes in *lbab/lbab *mice. Because the *lbab *mutation arose from PL/J, theoretically, there should be no difference in the *Nppc *sequence between *lbab *and PL/J mice except the mutation. In addition, this single nucleotide replacement is the only change between homozygous *lbab/lbab *and homozygous normal PL/J mice in 122 transcripts. However, we could not completely rule out the possibility that we may have missed other mutations/polymorphisms. Moreover, one could question whether this is a spontaneous polymorphism in mouse strains or a random mutation that arose after the *lbab *was separated from the PL/J. Therefore, we carried out two more experiments to further ensure the association between the mutation and the disease. First, we examined sequence polymorphism in exon 2 of *Nppc *from nine other inbred strains (Fig. 2C). As shown in Fig. 2D, each such mixture showed multiple bands, indicating the difference between *lbab/lbab *and those strains. Second, we bred 200 mice from heterozygous *lbab*/+ parents to evaluate allele frequency in relation to the phenotype. We genotyped every offspring by using the original pair of primers that flank exon 2 of the *Nppc *gene. From those progeny, we found 14 *lbab/lbab *mice that exhibited a genotype of homozygous G/G nucleotide and a phenotype of *lbab *mice, while the remaining progeny had 74 homozygous C/C and 112 heterozygous C/G genotypes, all with a normal phenotype. Taken together, our data indicate that the C→G transversion in exon 2 of the *Nppc *gene is associated with the phenotypes observed in *lbab *mice.

## Discussion

For the first time, we have identified a single nucleotide mutation by high throughput screening of a large genome region without fine mapping. The initial mapping at TJL was conducted with only 27 F_2 _animals. Linkage of *lbab *was first detected on Chr 1 with *D1Mit231 *and *D1Mit9 *by using the pooled sample. DNA samples were then typed for the individual 96 animals with these two markers and three additional Chr 1 markers [4]. By the standard strategy of classical positional cloning, the *lbab *locus could be further mapped. However, with the availability of mouse genome information and a tested protocol for high throughput screening of mutations [6,7], we directly searched genes based on the map from TJL. With the success of finding this mutation and others [6,7], we feel confident that we no longer need fine mapping for most mutations.

In this study, several lines of evidence indicate that a single nucleotide mutation of *Nppc *is associated with the *lbab *phenotype. First, *Nppc *is located within the genetic region of the *lbab *locus. Second, the *Nppc *mutation was the only defect detected among candidate genes and ESTs within the *lbab *locus from *lbab *mice. Because the *lbab *mutation was transferred from the PL/J strain to the B6 inbred strain, we evaluated the possibility of close linkages of nearby sequences from the PL/J mice by screening any sequence difference near the mutation area and later crossing with PL/J mice. There were no other differences between *lbab/lbab *mice and their two parental strains, so the possibility of other mutation involvement was ruled out. Third, cDNA sequence results agreed with the genomic DNA data. Last, we showed that the *Nppc *genotype is unique in *lbab/lbab *mice compared with nine other inbred strains, and the G/G *Nppc *genotype was closely associated with the phenotype in *lbab *mice.

Recently, several transgenic and knockout mouse studies have demonstrated that *Nppc *is critical in the prevention and rescue of achondroplasia [11,12]. A recent gene knockout study done by Chusho et al. [11] indicated that *Nppc *null mice of 129/Sv background showed severe dwarfism and early death. The *lbab *mice have a phenotype similar to *Nppc *knockout mice with two exceptions. First of all, the *lbab *mice develop an overall smaller body size. Second, the mutants exhibit proportionate dwarfing of all organs with the possible exception of the male reproductive tract, which appears extremely small [4]. However, much precise information may be obtained by a direct comparison between *Nppc *null mice and *lbab *mice. There may still be some difference between them because of the difference not only in the nature of the mutations but also in the genome backgrounds of those two models. Accordingly, we speculate that the identified point mutation of *Nppc *in *lbab *mice belongs to a loss-of-function mutation. As a key positive regulator of endochondral bone formation, *Nppc *seems to express its activity mainly through natriuretic peptide receptor 2 (Npr2) [11,13-15]. On the other hand, a recent study indicated that *Nppc *counteracts the activities of fibroblast growth factor signalling, which is a major negative regulatory pathway for long bone development, in both direct and indirect ways [16].

Murine *Nppc *is structurally similar to that of other species. The affected Arg at codon 117 on the *Nppc *domain is highly conserved among all members of the natriuretic peptide system and different species (Fig. 3B). The mechanisms for regulating Nppc expression are currently unknown. Importantly, the mutated nucleotide is also located in the common biologically active COOH-terminal 22 amino acid residue area, suggesting the critical significance of this amino acid residue in the functioning of these ligands during skeletal development.

Nppc was not in the list of candidate genes for the allelism test. According to the information on TJL webpage [4], allelisms tested were brachymorphic (bm) [17] with a ratio of disease/total of 0/42 progeny born, achondroplasia (cn) [18] with a ratio of 0/61 progeny born, osteochondrodystrophy (ocd) [19] with a ratio of 0/19 (4 unclassifiable) progeny born, and small (sml) [20] with a ratio of 0/59 progeny born. The first three loci are known to be located on Chr 19 [17], 4 [18], and 19 [19], respectively. The last one, sml, is either on Chr 6 or unknown [20]. Had *Nppc *been considered as a candidate gene, our initial screening would have been simpler, although we feel that some work is needed to exclude mutations in the nearby genes. In addition to the known function of *Nppc*, the fact that there is no other mutation in nearby genes in the *lbab *region is supporting evidence for the potential cause of the *lbab *phenotype by the single nucleotide mutation in *Nppc*.

Identifying the *Nppc *mutation in *lbab *mice provides useful information for human achondroplasia studies. It also demonstrates that, while candidate genes should be carefully examined based on gene function, it is feasible to identify mutated genes that are roughly mapped by linkage analysis by sequence-based positional candidate cloning strategy. We speculate that this strategy will be particularly useful for familial human diseases with small numbers of patients; in those cases, researchers usually have either a rough map or name/number of the chromosome of the disease locus. Furthermore, by using functional genomics and rodent models with spontaneous mutations yielding measurable phenotypes, we can rapidly identify mutational events in a cost-effective manner.

## Conclusion

In the present study, a sequence-based positional candidate cloning approach was applied to identify a gene mutation in *lbab *mice with abnormal endochondral ossification. Our results suggest that a single nucleotide mutation in gene *Nppc *is likely to be the causative factor and that the *lbab *mouse may be a useful model for human achondroplasia studies.

## Methods

### Mice

A heterozygous (*lbab/+*) breeding pair of mice was purchased from TJL, and a breeding colony was established at the research animal facility of the University of Tennessee Health Science Center (UTHSC). Experimental animal procedures and mouse husbandry were performed in accordance with the National Institutes of Health's Guide for the Care and Use of Laboratory Animals and approved by the UTHSC Institutional Animal Care and Use Committee.

### Genomic sequence information

Information about microsatellite markers and their locations were obtained from the Mouse Genome Informatics website [8] and the Ensembl mouse genome database [9]. The sequence data used in the paper were based on the information as of March 20, 2005.

### DNA and RNA

Genomic DNAs (gDNA) were extracted from the livers of phenotypically normal +/+ and *lbab/+ *mice, as well as from mutant *lbab/lbab *mice, by using a Qiagen Genomic-tip20/G (Qiagen, Alameda, CA) following the manufacturer's instructions. After determining the quality and quantity of the DNA in an Eppendorf photometer (Eppendorf Scientific, Westbury, NY), we used DNAs for large-scale mutational screening. Total RNAs were extracted from femurs by using Trizol reagent (Invitrogen, Carlsbad, CA), and the quality of the total RNA was checked by electrophoresis on a Spectronic Genesys spectrophotometer (Spectronic Instruments, Rochester, NY).

### High throughput screening

The gDNAs from *lbab/lbab*, *lbab*/+, and +/+ mice were used for temperature gradient capillary electrophoresis (TGCE) and sequence analysis using Reveal™ system (SpectruMedix; State College, PA). Based on the Ensembl and National Center for Biotechnology Information (NCBI) databases, primer pairs flanking the exons of known and predicted genes (including ESTs) within the *lbab *locus were designed using Primer3 software [10]. Primers were located approximately 100 bp 5' or 3' to each exon and, in general, produced 300–400 bp amplicons. For large exons that required multiple pairs of primers, primers were designed to allow neighboring DNA fragments to overlap each other by at least 50 bps. Primer pairs were synthesized from Illumina (San Diego, CA). PCR amplification of gDNA was performed in a 96-well plate format and consisted of 30–35 cycles at three temperatures: strand denaturation at 96°C for 30 sec, primer annealing at 54–60°C for 60 sec, and primer extension at 72°C for 120 sec. A TGCE device made by SpectruMedix was used to analyze amplicons from *+/+*, *+/lbab*, and *lbab/lbab *mice. The SpectruMedix system includes a high-throughput capillary electrophoresis instrument capable of analyzing 96 samples every 140 min. Heteroduplex analysis was subsequently performed using SpectruMedix software. Amplicons from *lbab *mice were sequenced if they differed from normal ones.

### Genotyping

A pair of primers flanking the position of a single nucleotide mutation of the *Nppc *gene (forward primer: CTCTTGGGTGCAGAGCTAGG; reverse primer: AGCTGGTGGCAATCAGAAAA) was used to genotype +/+, *lbab*/+, and *lbab/lbab *mice. The PCR products from heterozygous and homozygous mice were mixed with those from normal mice. The individual and mixed PCR products were then run on TGCE to detect possible sequence variations. PCR amplification was performed in a total volume of 25 μl at a final concentration of 1.5 mM MgCl_2 _concentration and 0.2 mmol/L each dNTP, 0.2 μmol/L oligonucleotide primer, 100 ng template DNA, and 0.7 units Taq polymerase (Fisher Scientific, Pittsburgh, PA) for 35 cycles of 94°C for 1 min, 50–55°C for 1 min, and 72°C for 1 min.

### RT-PCR amplification of *Nppc*

One-step RT-PCR kit (Invitrogen, Carlsbad, CA) was used to detect the expression of mutated *Nppc *at the mRNA level. Reactions were performed in a total volume of 50 μl with 8 ng/μl of total RNA, and 0.2 μM forward (AGCTGGTGGCAATCAGAAAA) and reverse (TCAGTGCACAGAGCAGTTCC) primers were used to amplify exons 2 to 3 of *Nppc*. First, cDNA synthesis and pre-denaturation were performed in single cycles at 50°C for 40 min and 94°C for 2 min. Next, PCR amplification was performed for 35 cycles: 94°C for 30 sec, 54–58°C for 36 sec, and 72°C for 2 min.

### DNA sequencing

DNA sequencing was conducted to verify the mutation in the gDNA and cDNA of *Nppc *from different species of mice. PCR products from both gDNA and cDNA were purified using an AMPure PCR Purification Kit (Agencourt, Beverly, MA), and the resultant products were sequenced using the BigDye^® ^Terminator v3.1 Cycle Sequencing Kit (Applied Biosystems, Foster City, CA). A total volume of 5 μl sequencing reactions including 2 μl Big Dye (plus Half-BD), 10–23 ng of purified DNA template, and 1–3 ρmoles of either forward or reverse universal sequencing primers was incubated for 37 cycles at 96°C for 180 sec, 50°C for 30 sec, and 60°C for 180 sec. Unreacted primers were removed by ethanol-acetate precipitation (3.75% 3 M NaOAc, 87.5% nondenatured 100% EtOH, and 8.75% dH_2_O, pH 4.6). The labeled products were dissolved in 0.02 mM EDTA in HiDi formamide prior to electrophoretically loading onto the SpectruMedix 96 capillary sequencing system. The same primers in the amplification of DNA fragments from either genomic DNA or mRNA were also used in the sequencing. Sequencing was conducted twice to verify the results for both gDNA and cDNA.

### Statistical analysis

The body weight and body length for individual mice were measured at an average age of 5.5 days after birth. The weight and length differences between normal and *lbab *mice were analyzed for their statistical significance by using two-tail t-test with a cutoff p value of < 0.05.

## Authors' contributions

YJ carried out the majority of the molecular work including DNA and RNA isolation, primer design, RT-PCR, genotyping, sequencing, and data analysis.

JY drafted the paper and provided helpful comments on experimental execution and data analysis.

FJ provided animal samples and performed TGCE running.

HY did the original screening.

LRD, XL, BAR, and JS provided valuable input and help in drafting the paper.

WG initiated and mentored the study, as well as provided the valuable framework to draft the paper.

All authors read and approved the final manuscript.
